# Diagnosis and treatment of a dural arteriovenous fistula presenting with progressive parkinsonism and dementia: A case report and literature review

**DOI:** 10.3892/etm.2014.2122

**Published:** 2014-12-09

**Authors:** CHEN MA, QIAOLI LU, WANCHAO SHI, ZHIGUO SU, YUJUN ZHAO, CHEN LI, ZHENLIN LIU

**Affiliations:** 1 Department of Neurology, The Fifth Central Hospital of Tianjin, Tianjin 300450, P.R. China; 2 Department of Neurosurgery, The Fifth Central Hospital of Tianjin, Tianjin 300450, P.R. China

**Keywords:** dural arteriovenous fistula, parkinsonism, dementia, digital subtraction angiography, transarterial embolization

## Abstract

A dural arteriovenous fistula (DAVF) presenting with parkinsonism and dementia is rare; thus, is easily misdiagnosed. The present study reports the case of a 62-year-old male with mobility disabilities and a cognitive disorder. The initial symptoms were progressive symmetrical limb stiffness and weakness without significant limb tremor, and subsequently the appearance of progressive memory loss, behavioral abnormalities and a decline in the activities of daily living. Cranial magnetic resonance imaging (MRI) revealed an enlarged vascular shadow at the meninges of the left temporal lobe. In addition, digital subtraction angiography (DSA) revealed a DAVF in the left temporal region, fed by the bilateral middle meningeal arteries and meningeal branches of the vertebral artery, which were enlarged abnormally, with poor venous reflux to the superior sagittal sinus. The patient was treated with transarterial embolization therapy. Intraoperative angiography showed almost complete embolization of the DAVF. At day 3 following the surgery, the muscle tension of the bilateral limbs decreased significantly. After two weeks, the memory ability of the patient had recovered to the level prior to the onset, and the gait was stable. At one month post-surgery, the patient was able to take care of himself completely, and after three months, a stereotactic treatment was conducted for the residual fistula. At the one year follow-up, neurological examination revealed that the patient had recovered normally. In conclusion, progressive parkinsonism and dementia with an abnormal flow void shadow on cranial MRI films should be considered as a possible diagnosis of a DAVF. In these cases, DSA and endovascular treatment are recommended as soon as possible.

## Introduction

A dural arteriovenous fistula (DAVF) refers to an abnormal direct blood connection between an intracranial artery and the dural venous sinus, and accounts for 10–15% of all intracranial vascular malformations (1). It is known that there are congenital and acquired causes of DAVF. The majority of cases of DAVF are congenital and caused by dural vascular abnormalities. However, certain cases of DAVF are acquired and may be caused by brain trauma, venous sinus inflammation, venous sinus thrombosis formation, brain surgery, hypercoagulable states and others. DAVFs most commonly occur in the cavernous sinus, transverse sinus, sigmoid sinus and superior sagittal sinus. The main treatment for DAVF is endovascular embolization (2). The principle of DAVF treatment is the ‘permanent and complete occlusion of the fistula’. However, in certain cases (including cortical venous reflux disease DAVF and wide DAVF) the DAVF cannot be completely cured, so the goal of treatment changes to reduce the rate of bleeding and relieve clinical symptoms. Treatments for DAVF include endovascular embolization, vascular compression, surgery and stereotactic treatment. With the development of modern materials and devices, endovascular treatment has become the main method for DAVF treatment (2). Endovascular interventional embolization treatment includes transartery embolization, transvenous embolization and the united arteriovenous approach. The main clinical manifestations of DAVF depend on the position of the venous drainage, drainage direction, velocity and the position of the fistula. DAVF manifestations of ophthalmic vein drainage are headaches, exophthalmos and conjunctival congestion; cortical venous drainage in patients is usually manifested as local neurological dysfunction and brain hemorrhage (3). A previous study observed that lesions in the craniocervical junction region, frequently to the brain stem vein, and cervical spinal perimedullary venous drainage revealed subarachnoid hemorrhage (4). DAVFs have various clinical manifestations, but rarely present as parkinsonism and dementia. Therefore, DAVFs are easily misdiagnosed. The present study reports the case of a DAVF manifesting as parkinsonism and dementia, and reviews the relevant literature.

## Case report

Written informed consent was obtained from the patient’s family for inclusion in the present case report. A 62-year-old male was admitted to hospital after presenting with progressive limb stiffness and weakness for five months, as well as memory loss and unstable walking for two weeks. Five months prior to admission, the patient developed limb stiffness and weakness with no incentive, particularly when going downhill. In addition, the patient exhibited slow movements, which sequentially involved the upper limbs, but without an evident tremor. No treatment was administered during this period. At two weeks prior to admission, the patient presented with aggravated limb stiffness and weakness, walking instability (rushing forward and difficulty stopping when walking), memory loss (the performance of forgetting commonly used figures and short-term memory loss), apathy and urinary incontinence. The patient had been hospitalized at a local hospital and was diagnosed with cerebral infarction and Parkinson’s disease; however, no treatment was administered after improving the circulation and anti-Parkinson therapy. Thereafter, the patient was transferred to the Tianjin Fifth Central Hospital (Tianjin, China) for further diagnosis and treatment.

**Figure 1:**
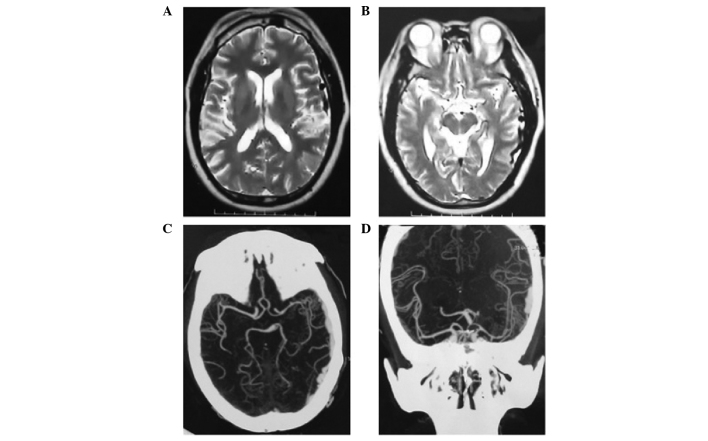
Preoperative cranial MRI and CTA scans. Preoperative cranial MRI scans revealed (A) a flow void shadow of enlarged meningeal vessels in the left temporal lobe and (B) a vermiform flow void shadow of vessels in the left temporal lobe. (C) Brain CTA showed multiple vermiform enlarged vessels in the left cerebral hemisphere. (D) Coronal CTA revealed multiple vermiform enlarged vessels in the left cerebral hemisphere and the right frontoparietal lobe. MRI, magnetic resonance imaging; CTA, computed tomography angiography.

The patient had a history of hypertension for 10 years, coronary heart disease and atrial fibrillation for five years, and had undergone coronary stenting, but had no history of encephalitis, traumatic brain injury or poisoning. On examination, the patient exhibited a blood pressure of 150/80 mmHg, an irregular heartbeat, clear consciousness, apathy, slurred speech, disorientation, memory loss and decreased comprehension and calculation abilities. The patient scored 11 points on the mini-mental state examination. Muscle strength in four limbs was slightly lower than normal with limb hypermyotonia, particularly in the lower limbs. Babinski’s sign (−) was observed on both feet. The patient walked forward with fewer movements and postural instability, and was classified with Hoehn-Yahr stage III of Parkinson’s disease. Cranial magnetic resonance imaging (MRI) revealed long T1 and T2 signals in the bilateral frontal lobes, a high FLAIR signal, an enlarged vascular shadow at the meninges of the left temporal lobe and mild cerebral atrophy (Fig. 1A and B). Computed tomography angiography (CTA) revealed multiple vermiform enlarged vessels on the left cerebral hemisphere and the right frontal and parietal brain surface, considered to be a left temporal DAVF (Fig. 1C and D). Furthermore, an electroencephalography revealed frontotemporal slow waves, and a lumbar puncture examination showed normal pressure in the cerebrospinal fluid, which was l40 mm H_2_O, while the cerebrospinal fluid cellular and biochemical tests were normal.

**Figure 2:**
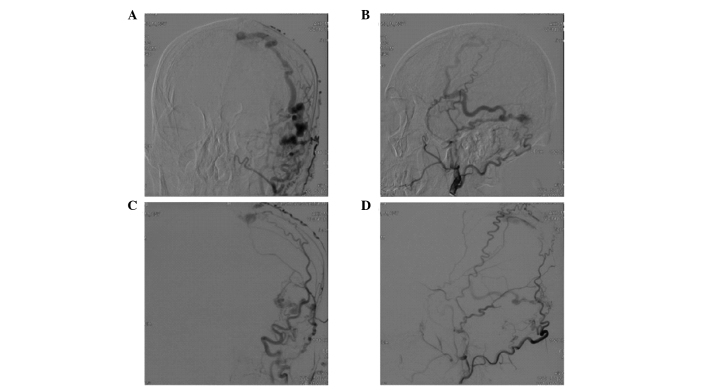
Preoperative and postoperative DSA images. (A and B) Preoperative anterioposterior and lateral DSA images revealed an abnormally enlarged middle meningeal artery serving as feeders with venous reflux to the superior sagittal sinus. (C and D) Postoperative anterioposterior and lateral DSA images revealed a dural arteriovenous fistula that was almost fully embolized, with the venous phase revealing a markedly decreased reflux. DSA, digital subtraction angiography.

The patient was initially diagnosed with parkinsonism, vascular dementia and a DAVF. Digital subtraction angiography (DSA) revealed a DAVF in the left temporal region, fed by the bilateral middle meningeal arteries and meningeal branches of the vertebral artery, which were enlarged abnormally, with poor venous reflux to the superior sagittal sinus (Fig. 2A and B). The patient was treated with transarterial embolization therapy. Following general anesthesia, a Malathon microcatheter was inserted into the left branch of the middle meningeal artery, guided by a Mirage 0.008 microwire, and the tip of the microcatheter was close to the fistula. Thereafter, Onyx-l8 glue (~1.5 ml), was slowly injected for embolization. Intraoperative angiography showed almost complete embolization of the DAVF, and the venous reflux was slower than before (Fig. 2C and D). At day 3 following surgery, the condition of the patient improved. In comparison with the preoperative conditions, the muscle tension of the bilateral limbs was significantly decreased, movement during walking was improved, the walking start and leg speed were faster and turn-back and cognitive function had improved. In addition, at two weeks post-surgery, the memory ability of the patient had recovered to the level prior to onset, and the gait was stable. Scores of the mini-mental state examination were up to 25 points, and the Hoehn-Yahr level was stage I. At discharge, the patient was diagnosed with a left temporal DAVF.

## Discussion

DAVFs can be developed at all ages, but are most common in individuals aged between 60 and 70 years. The ratio of females to males with a DAVF is 1:1.65 (5). DAVFs are mainly fed by the external carotid artery; however, the internal carotid and meningeal branches of the vertebral artery can be involved. In the present case, the branches of the external carotid artery and meningeal branches of the vertebral artery fed the DAVF. Venous sinus hypertension is considered to be the leading cause of an acquired DAVF (6). A physiological arteriovenous access exists between the network of dural arteries and the dural sinus. Under venous hypertension, the physiological arteriovenous channels around the venous sinus remain open, and a pathological arteriovenous shunt forms and attracts a large number of middle meningeal arteries involved in the blood supply through enriched tiny arteries from the dural wall, forming a DAVF (7,8). Poor reflux of the superior sagittal sinus in the present case may be the initiating factor of the temporal DAVF.

DAVFs are often involved in the transverse sinus and sigmoid sinus, followed by the cavernous sinus and superior sagittal sinus, but are rarely found in the straight sinus, with complex and diverse clinical manifestations, but no significant specificity. In the present case, the symptoms of the DAVF were progressive parkinsonism and dementia, which is rare and has been rarely reported (9,10). The initial symptoms were progressive limb stiffness and weakness with symmetrical onset; however, there was no significant limb tremor, abnormal posture and difficulty in starting to walk. Soon afterwards, the patient presented with progressive memory loss, behavioral abnormalities and a decline in activities of daily living, which were more in line with the clinical diagnosis of Parkinson’s disease and dementia. A number of pathophysiological mechanisms were considered to cause the present case of DAVF, which resulted in parkinsonism and dementia. Firstly, the direct connection between the arterial fistula and the superior sagittal sinus caused a partial arterial steal phenomenon that led to frontal, temporal lobe and basal ganglia ischemia, among which, frontal white matter and basal ganglia damage triggered parkinsonism (11), while dementia was generated from the involvement of the frontal and temporal lobe. Secondly, localized venous hypertension, venous congestion and decreased cerebral perfusion due to abnormal venous return caused local ischemia and hypoxia, which resulted in the release of vascular endothelial growth factor and induced angiogenesis. Finally, parkinsonism and dementia may have been the result of cerebral compression by dilated veins in the frontotemporal area.

Due to varying nonspecific clinical manifestations, the diagnosis of a DAVF is often based on imaging studies (12). Diagnosis is difficult using cranial CT; however, this imaging technique can reveal certain secondary changes caused by a DAVF, including venous sinus thrombosis, acute and sub-acute subarachnoid hemorrhage and subdural or cerebral hemorrhage. However, cranial MRI is better compared with CT for the detection of DAVF-induced secondary changes, and is useful to show the extensive flow void phenomenon (Fig. 1A). In addition, with a serious condition, MRI can display tortuously enlarged cortical veins (Fig. 1B). In the present case, the patient was misdiagnosed prior to admission to the Tianjin Fifth Central Hospital. An abnormal flow void area was identified in the left temporal lobe from the cranial MRI films, and subsequently CTA and DSA were essential for the final diagnosis. CTA and MRI show abnormal enlarged feeding arteries, dilated veins and dural sinuses, but are unable to reveal the situation of the fistula or the existence of potential anastomosis and small feeding arteries. DSA is better for demonstrating the characteristics of a DAVF, including the site of the fistula, feeding features and venous return, and even can indirectly demonstrate the degree of fistula blood flow and intracranial hemodynamic characteristics (13).

The treatment methods for DAVFs include embolization, vascular compression, surgery and stereotactic treatment. In addition, with the invention of novel materials and devices, endovascular therapy has become the primary method for DAVF treatment. The treatment principle is to occlude the dural venous fistula. Theoretically, it is better to directly embolize the fistula by transvenous embolization, as this is easier to achieve an anatomic cure. The arterial approach is a substitute for the intravenous approach, and is used for patients without venous sinus drainage, with sinus stenosis or if the fistula is simply fed by the branches of the external carotid artery or the non-tortuous enlarged feeding artery (14). Onyx glue is often selected as the embolic material since it exhibits excellent dispersibility, is not easily broken up or adheres to a microcatheter and is easy to control. The maximum degree of embolization can be obtained with curative outcomes (15). In the present case, a microcatheter was inserted into the fistula of the middle meningeal artery and Onyx-l8 glue was slowly injected, ultimately achieving embolization. Following surgery, the symptoms of parkinsonism and dementia were significantly reduced, and the neurological function and scores on the dementia scale showed a substantial increase over the preoperative evaluation. After one month, the patient was able to take care of himself completely, and after three months, a stereotactic treatment was performed for the residual fistula. At the one year follow-up, neurological examination revealed that the patient was recovering normally.

In conclusion, a DAVF presenting as parkinsonism and dementia is easily misdiagnosed. However, a timely diagnosis is crucial for improved therapeutic outcomes. In cases of rapidly progressive parkinsonism or dementia, accompanied by an abnormal flow void shadow on cranial MRI films, the possibility of a DAVF should be considered. Subsequently, the DAVF should be treated with appropriate and timely selective endovascular treatment, with DSA recommended as soon as possible.
